# Hyperlocal Postcode Based Crowdsourced Surveillance Systems in the COVID-19 Pandemic Response

**DOI:** 10.3389/fpubh.2020.00286

**Published:** 2020-06-09

**Authors:** Ajay Hegde, Ramesh Masthi, Darshan Krishnappa

**Affiliations:** ^1^Senior Clinical Fellow, Queen Elizabeth University Hospital, Glasgow, United Kingdom; ^2^Professor Head of Community Medicine and Public Health, Kempegowda Institute of Medical Sciences, Bangalore, India; ^3^Department of Medicine, University of Minnesota Medical School, Minneapolis, MN, United States

**Keywords:** COVID-19, Pandemic response, post code map, digital health, surveillance, crowdsource

## Abstract

The SARS-CoV-2 pandemic has rapidly saturated healthcare resources across the globe and has led to a restricted screening process, hindering efforts at comprehensive case detection. This has not only facilitated community spread but has also resulted in an underestimation of the true incidence of disease, a statistic which is useful for policy making aimed at controlling the current pandemic and in preparing for future outbreaks. In this perspective, we present a crowdsourced platform developed by us for the true estimation of all SARS-CoV-2 infections in the community, through active self-reporting and layering other authentic datasets. The granularity of data captured by this system could prove to be useful in assisting governments to identify SARS-CoV-2 hotspots in the community facilitating lifting of restrictions in a controlled fashion.

In 2018, The Centers for Disease Control and Prevention (CDC), USA evaluated the preparedness to respond to future pandemics, in comparison to the 1918 Spanish Influenza Pandemic. It highlighted that a century worth of advances in influenza surveillance through WHO network laboratories, rapid diagnostics, vaccination programmes and antiviral treatment in addition to improvements in government and health care systems, may not be sufficient to control a large scale pandemic (1). The severe acute respiratory syndrome coronavirus 2 (SARS-CoV-2) infection and the associated coronavirus disease 2019 (COVID-19) has vindicated their assessment and has assumed pandemic proportions affecting individuals in over 190 countries across all continents except Antarctica (2).

Rapid global spread of the infection has led to an acute strain on the healthcare infrastructure, resulting in a shortage of healthcare equipment and overburdening of an already overworked healthcare workforce (3). The situation is compounded in under-developed and developing countries, exposing the fragile healthcare ecosystems in these countries. Public health measures are currently focused on “flattening the curve” to slow the rate of infection and ease the acute stress on healthcare systems. User interface (UI) experts are creating graphical depictions of the impact of the different strategies employed by various countries across the world on the spread of virus, within their respective communities (4).

While the World Health Organization (WHO) has advocated for extensive testing to identify and isolate infected individuals, the large population that needs screening has led to a rapid depletion of test kits (5). Fluid testing criteria with the lack of widespread testing has led to an under detection of the infected people (6) and may not estimate the true extent of community infection. The community mitigation measures practiced during outbreaks in the 20th Century, now rephrased as social distancing and lockdowns have become the norm in most countries as we write this article. Such lockdowns, however, have adverse economic and social consequences, both in the short and long term.

While nationwide lockdowns, have helped control the infection, the negative socio-economic consequences are far reaching. Governments across the world are now reconsidering such restrictions, further emphasizing the need to harness technology in the identification of potential hotspots of case clusters (syndromic surveillance for influenza like illness). This can result in direct smaller scale “mass” isolation which would be a lesser strain on the economic and social health of countries. With this in mind, countries like China and South Korea have adopted stringent surveillance measures. Though useful in such emergencies such technology can results in a significant breach of privacy with a potential for long term misuse (7).

Back in 1854, John Snow, an English physician meticulously mapped the cluster of cholera cases centered around a hand pump in Broad (now Broadwick) Street, London and proved waterborne transmission of the disease (Figure 1a). He used government death-registration data and house-to-house enquiries to map the victims' residences, showing their proximity to the pump (8). Today 166 years later, with widespread internet access, smartphones equipped with Global Positioning System (GPS), Geographic Information system (GIS), Artificial Intelligence and Big Data technologies, we haven't been able to generate regional maps with such fine details of case to case transmission for the Covid-19 pandemic. One handy dashboard developed by the Johns Hopkins University, Baltimore, has been providing valuable statistics primarily reported by government bodies across the globe (9). Though extremely useful at communicating the global pandemic, these dashboards provide little information on regional distribution within communities. In such scenarios crowdsourced surveillance systems involving the active participation of the general population with self-reporting of health conditions and disease symptoms are an attractive option and have been used with some merit in previous disease outbreaks (10). Real-time tracking of infectious diseases, is challenging and still not a priority in developing countries (11), and the potential of crowdsourcing technologies are yet to be tapped.

**Figure 1:**
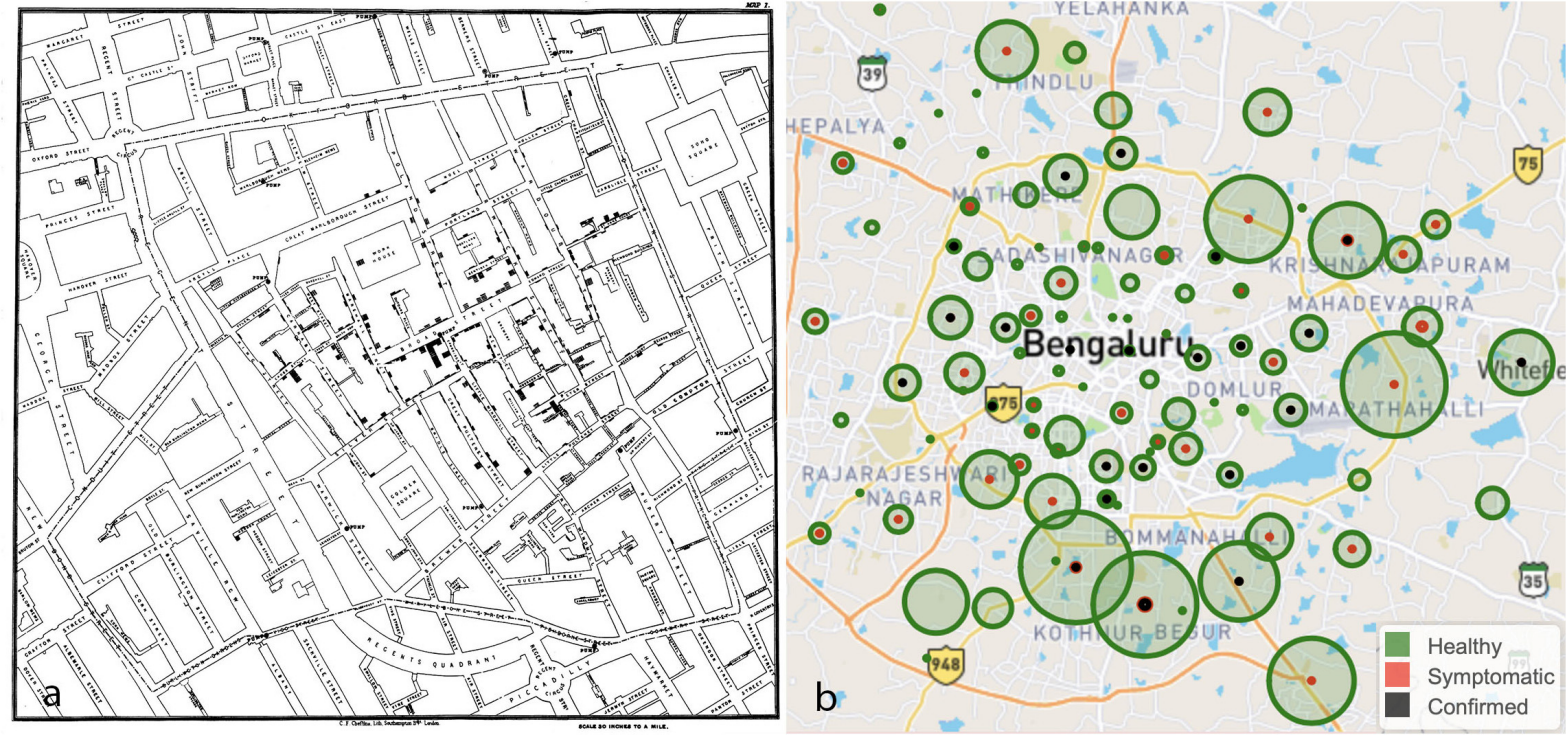
**(a)** The map by John Snow showing the clusters of cholera cases in the London epidemic of 1,854 around Broad Street. **(b)** A crowdsourced map of Bengaluru, a city in India depicting healthy (green), symptomatic (red) and confirmed positive (black) individuals. Source: https://www.trackcovid-19.org.

Having monitored this space over the last 2 months, we as health professionals conceptualized and created a crowdsourced symptom tracker to capture influenza like illness on a map with the granularity of a postcode. Users can self-report the presence or absence of common symptoms associated with the SARS-CoV-2 infection and probable exposure to a SARS-CoV-2 positive patient in addition to demographics which include age, sex, and Postcode. An email address is optional to receive updates. Further, we also scan the internet and open-source COVID-19 databases daily for postcodes or locations of confirmed positive cases to include in the website in realtime (12). In populous countries, field workers can also provide this valuable real-time data to halt transmission in nascent stages. Finally, we layer all this data, converting postcodes to latitude and longitude using the Google GeolocateAPI, on to a map, using the Leaflet Maps API (Figure 1b). By layering both crowdsourced symptom data and confirmed positive cases, case to case transmission can be established. We chose postal code as the identifier to map individuals as this is the most consistent, reproducible addressograph that can be obtained. However, the average area covered by a postcode varies from 10 square miles in the United Kingdom (UK) to 32 square miles in India and 90 square miles in the United States of America (USA).

Such crowdsourced information will help identify hot spots and the distribution of potentially infected individuals in a given postcode location. It provides trends of affected people in a given city which can be used by public health organizations to focus preventive, isolation and treatment efforts toward containment of illness thus enabling governments to open up communities in a controlled fashion, minimizing economic damage. With the pandemic moving into the phase of community transmission, such applications which focus on syndromic surveillance generate more valuable data than digital contact tracing technologies which have been rolled out in several countries across the globe. However, it would not provide individual risk stratification based on the level of exposure.

Poor engagement will be the most significant limiting factor, of such crowdsourced platforms which have excellent potential as surveillance tools in this digital era. Although these systems may not elicit a good response from the general population during seasonal influenza outbreaks, the magnitude of the COVID-19 pandemic and the consequences of containment measures employed so far can make it socially more acceptable. Local governments will need to play an essential role in strengthening such a system and advising their citizens accordingly. Another limitation of this system can also be the misreporting of information which would lead to misinterpretation of cases. Users will be skeptical about privacy, data protection, compliance, and safeguarding these will be the highest priority. Our system does not capture any identifiable personal information which can be traced back to the user.

We hope this rapidly prototyped, lean application, developed in lines with the startup culture of the Silicon Valley, will at least alleviate if not solve some problems during the grim times of the SARS-CoV-2 pandemic. Active participation from public health units across the world in sharing data and implementing out of the box technologies will yield results in geofencing the SARS-CoV-2 infection.

The platform can be accessed at https://www.trackcovid-19.org.

## Data Availability Statement

Publicly available datasets were analyzed in this study. This data can be found here: https://www.trackcovid-19.org.

## Author Contributions

AH was involved in design of system and preliminary write up. RM provided public health validation and revision of manuscript. DK was involved in drafting the article and revisions. All authors contributed to the article and approved the submitted version.

## Conflict of Interest

The authors declare that the research was conducted in the absence of any commercial or financial relationships that could be construed as a potential conflict of interest.
